# TLR8 gain-of-function syndrome

**DOI:** 10.70962/jhi.20260172

**Published:** 2026-10-22

**Authors:** Hwi M. Gil, Megan A. Cooper

**Affiliations:** 1 Division of Rheumatology/Immunology, Department of Pediatrics, Washington University School of Medicine, St. Louis, MO, USA

## Abstract

Toll-like receptor 8 (TLR8) is an endosomal pattern recognition receptor highly expressed in myeloid cells that senses RNA degradation fragments. Monogenic gain-of-function (GOF) variants in *TLR8* cause an inborn error of immunity (IEI) with a spectrum of immune-mediated pathology ranging from severe lymphoproliferation with neutropenia and bone marrow failure to anemia and autoimmunity. Half of identified patients have mosaic disease-causing variants, with variant allele fractions as low as 7% sufficient to drive systemic immune dysregulation. This, along with immunologic profiling of patients, demonstrates a dominant mechanism of disease, likely mediated by inflammatory cytokines. Disease-causing variants in *TLR8* are enriched at or near agonist and antagonist binding sites in the encoded protein. As a spectrum of disease and genotypes emerges, identifying sensitivity to agonism and resistance to antagonism may provide insight into molecular mechanisms and therapeutic targets. Here, we review the genetics and clinical features of 19 reported cases of TLR8 GOF diseases, and future areas of investigation for this IEI.

## Teaser

In this Review, Gil and Cooper review the molecular, immunologic, and clinical features of TLR8 gain-of-function syndrome. They review findings from patients with mosaic and germline disease, and integrate knowledge of key structural components of the TLR8 protein.

## Introduction

Toll-like receptors (TLRs) are type I transmembrane pattern recognition receptors that are a critical part of innate immunity against infection or injury (1). TLRs sense pathogen-associated molecular patterns (PAMPs) and damage-associated molecular patterns (DAMPs), and in response promote proinflammatory gene expression and cytokine production (1). There are 10 human TLRs which can be grouped by those localized on the cell surface (TLR1, TLR2, TLR4, TLR5, TLR6, TLR10) and those in intracellular compartments such as the endosome or endoplasmic reticulum (TLR3, TLR7, TLR8, TLR9) (1, 2). TLRs function as homo- or heterodimers, with each monomer containing a horseshoe-shaped ectodomain of multiple leucine-rich repeats (LRRs), a transmembrane domain, and a cytoplasmic Toll/IL-1 receptor (TIR) domain (1, 2).

Defects in genes encoding several TLRs have been linked to monogenic inborn errors of immunity (IEIs) (3, 4). Loss-of-function (LOF) variants in nucleic acid–sensing TLRs can cause pathogen-specific susceptibility. TLR3 deficiency was first identified as causing susceptibility to herpes simplex encephalitis (5) and subsequently to severe influenza pneumonia (6). More recently, LOF variants in both *TLR3* and *TLR7* have been linked to severe COVID-19 (7, 8, 9). Biallelic LOF variants in *TLR4* were described with a variable phenotype associated with impaired sensing of bacterial-derived lipopolysaccharides (10).

We originally reported 6 patients with gain-of-function (GOF) variants in *TLR8* associated with neutropenia, lymphoproliferation, and bone marrow (BM) failure in 2021 (11). Since that time, 13 additional cases of TLR8 GOF have been identified (Table 1, summary of published TLR8 GOF patients) (11, 12, 13, 14, 15, 16, 17, 18). Recently, *TLR7* GOF was identified as an IEI associated with susceptibility to monogenic systemic lupus erythematosus, with a phenotypic spectrum broadened beyond autoimmunity, with some hematologic abnormalities overlapping with TLR8 GOF syndrome including anemia, thrombocytopenia, and lymphopenia (19, 20, 21, 22, 23, 24, 25, 26, 27). Multiple other IEIs are caused by defects upstream and downstream of the TLR signaling pathway, for example, in genes encoding MyD88, IRAK4, IKBα, NEMO, and *UNC93B1*, highlighting the nonredundant importance of TLR signaling in human immunity (3, 4, 28, 29, 30, 31, 32, 33).

**Table 1.** Reported TLR8 GOF patients

#	Ref ID	Sex	Age	Variant	Site affected	Mosaic or germline	VAF	Major immunophenotype/clinical symptoms	TLR8 function and/or expression	Treatment	Reported outcome	Ref
**INFLTR8/neutropenia with BM involvement**												
1	P1	M	15 years	p.P432L	Antagonist binding site	Mosaic	14%	**Heme**: neutropenia, thrombocytopenia, anemia, LGL, HSM, liver hemophagocytosis **Imm**: low IgG, ↑ serum cytokines, inverse CD4:CD8 ratio, ↑ TEMRAs **BM**: lymphoid aggregates, clonal LGL, BM failure **Other:** infections, oral ulcers, fever	• NF-κB reporter assay	G-CSF, IVIg corticosteroids, sirolimus	Deceased	Aluri et al. (11) and Arnold et al. (12)
2	P2	M	9 mo	p.P432L	Antagonist binding site	Mosaic	18%	**Heme:** neutropenia, anemia, HSM **Imm:** low IgG, ↑ serum cytokines **BM:** agranulocytosis **Other:** infections, fever	• Recurrent variant • TLR8 stimulation–dependent phospho-NF-κB and cytokine production in iPSC-derived neutrophils and macrophages	G-CSF, IVIg, corticosteroids, sirolimus, anakinra, HCT	Deceased s/p 2 × HCT	Aluri et al. (11) and Arnold et al. (12)
3	P3	M	7 years	p.F494L	Antagonist binding site	Mosaic	8%	**Heme:** neutropenia, thrombocytopenia, HSM **Imm:** low IgG, ↑ serum cytokines, inverse CD4:CD8 ratio, ↑ TEMRAs **BM:** hypocellular, myeloid hypoplasia, lymphohistiocytic aggregates, TCRγ/δ and CD8^+^ T cell infiltration **Other:** infections, oral ulcers	• NF-κB reporter assay • TLR8 stimulation–dependent cytokine production in iPSC-derived neutrophils and macrophages	G-CSF, corticosteroids, sirolimus, HCT	Well s/p HCT	Aluri et al. (11) and Arnold et al. (12)
4	P4	M	16 years	p.P432L	Antagonist binding site	Mosaic	26%	**Heme:** neutropenia, thrombocytopenia, anemia, HSM, T-LGLL **Imm:** low IgG, B cell deficiency, inverse CD4:CD8 ratio, ↑ TEMRAs **BM:** agranulocytosis, CD8^+^ T cell infiltration, clonal LGL **Other:** infections	• Recurrent variant	G-CSF, IVIg, corticosteroids, alemtuzumab, HCT	Well s/p HCT	Aluri et al. (11) and Arnold et al. (12)
5	P5	M	5 years	p.P432L	Antagonist binding site	Mosaic	17%	**Heme:** neutropenia, thrombocytopenia, anemia, LAD, HSM **Imm:** low IgG, ↑ serum cytokines **BM:** T cell infiltration, myeloid hypoplasia **Other:** infections, oral ulcers, fever, type I diabetes, GI tract inflammation	• Recurrent variant	G-CSF, IVIg, corticosteroids, sirolimus, WinRho, MMF, HCT	Well s/p HCT	Aluri et al. (11) and Arnold et al. (12)
6	P6	M	9 mo	p.G572D	Site 1	Germline *de novo*	NR	**Heme:** neutropenia, thrombocytopenia, anemia, LAD, HSM **Imm:** low IgG, ↑ serum cytokines, inverse CD4:CD8 ratio **BM:** myeloid hypoplasia, BM failure **Other:** infections, fever	• NF-κB reporter assay	G-CSF, IVIg, antibiotics, pRBC, rituximab, MMF	Deceased	Aluri et al. (11) and Arnold et al. (12)
7	P7	M	9 years	p.P432Q	Antagonist binding site	Mosaic	7%	**Heme:** neutropenia, thrombocytopenia, anemia, LGL, HSM **Imm:** ↑ CD8^+^ clone **BM:** CD8^+^ T cell infiltration, inverse CD4:CD8 ratio, clonal LGL **Other:** infections, oral ulcers, arthritis	• NF-κB reporter assay	G-CSF, steroids, rituximab HCT	Deceased d15 after HCT	Arnold et al. (12)
8	P8	M	10 years	p.P432L	Antagonist binding site	Mosaic	12%	**Heme:** neutropenia, anemia, HSM, LGL **Imm:** inverse CD4:CD8 ratio, ↑ TEMRAs, ↑ CD4^+^ T central memory **BM:** CD8^+^ T cell infiltration, clonal LGL, myeloid hypoplasia **Other:** oral ulcers, pneumonia	• Previously characterized by Aluri et al. (11)	G-CSF, steroids, tofacitinib, hydroxychloroquine, HCT	Well s/p HCT	Arnold et al. (12)
9	P9	M	28 years	p.P432R	Antagonist binding site	Mosaic	15%	**Heme:** neutropenia, thrombocytopenia, anemia, HSM, T-LGLL **Imm:** NR **BM:** CD8^+^ T cell infiltration, clonal LGL **Other:** oral ulcers, pneumonia	• NF-κB reporter assay	G-CSF, prednisone, azathioprine, cyclophosphamide, MTX, rituximab	Lost to f/u	Arnold et al. (12)
10	P10	F	0.5 mo	p.P432L	Antagonist binding site	Germline *de novo*	50%	**Heme:** neutropenia, anemia, HSM **Imm:** low IgG, low CD27+IgD class-switched memory B cells **BM:** erythroid hypoplasia **Other:** urinary tract infections	• Previously characterized by Aluri et al. (11)	IVIg, abatacept, HCT	Well s/p HCT	Arnold et al. (12)
11	n/a	M	8 years	p.L433F	Antagonist binding site	Germline inherited	100%	**Heme:** neutropenia, pancytopenia, HSM **Imm:** low IgG, ↑ inflammatory markers, inverse CD4:CD8 ratio, B and NK cell lymphopenia **BM:** HLH, T cell infiltration **Other:** oral ulcers, infections, rash, mother affected (#19 here)	• NF-κB reporter assay	Dexamethasone, etoposide, emapalumab, HCT	Well s/p HCT	Barber et al. (18)
12	n/a	M	17 years	p.G572D	Site 1	Germline *de novo*	100%	**Heme:** neutropenia, AIHA **Imm:** NR **BM:** autoimmune myelofibrosis, CD3^+^ T cell lymphoid hyperplasia **Other:** oral ulcers, infections	• Previously characterized by Aluri et al. (11)	Corticosteroids, thalidomide, tofacitinib	Persistent neutropenia	Qu et al. (13)
13	n/a	F	50s	p.F494Y	Antagonist binding site	Mosaic	12%	**Heme:** neutropenia, LGL **Imm:** low IgG, ↑ serum cytokines, ↑ DNT cells, B cell deficiency **BM:** CD8^+^ T cell infiltration **Other:** pneumonia	• NF-κB reporter assay	GM-CSF, M-CSF, MTX, cyclosporine, JAKi + IVIg	Stable on therapy	Patel et al. (14)
**Anemia ± inflammation and autoimmunity**												
14	A	M	6 years	p.G572V	Site 1	Germline inherited	NR	**Heme:** AIHA, HSM, LAD **Imm:** low IgG, ↑ inflammatory markers, reduced Th1, reduced effector memory and terminally differentiated CD8^+^ T cells **BM:** NR **Other:** infections, oral ulcers, panniculitis	• NF-κB reporter assay • TLR8 stimulation–dependent NF-κB and cytokine production in PBMCs and monocytes • TLR8 protein expression ∼50% in monocytes and granulocytes • Hypersensitivity to TLR7 stimulation • Accelerated TLR8 protein degradation in HEK293T cells	IVIg, corticosteroids, azathioprine, rituximab, sirolimus, hydroxychloroquine	Well	Fejtkova et al. (15)
15	B	M	2.5 years	p.G572V	Site 1	Germline inherited	NR	**Heme:** AIHA, HSM, LAD, vasculitis (CNS) **Imm:** low IgG, reduced effector memory and terminally differentiated CD8^+^ T cells **BM:** NR **Other:** infections, oral ulcers, polyarthritis, enteropathy, erythema nodosum		IVIg, corticosteroids, MTX, azathioprine, rituximab, anakinra, HCT	Well s/p HCT	Fejtkova et al. (15)
16	P1	M	<1 year	p.A518T	Site 1/antagonist binding site	Germline inherited	NR	**Heme:** anemia, thrombocytopenia, leukopenia, LAD, splenomegaly, vasculitis (cerebral and polyarteritis nodosa) **Imm:** low IgG, ↑ serum cytokines, T cell lymphopenia (P2), reduced memory B cells **BM:** normal erythropoiesis and thrombopoiesis **Other:** infections, fever, intellectual disability	• Phospho-NF-κB at baseline in patient monocytes • TLR8 protein expression ∼50% in ectopically expressed HEK Null1 cells and BLaER1 cells • Accelerated proteasomal degradation of TLR8	IVIg, corticosteroids, cyclophosphamide, azathioprine, mycophenolic acid, infliximab	Well on therapy	Skenteris et al. (16)
17	P2	M	<1 year	p.A518T	Site 1/antagonist binding site	Germline inherited	NR			IVIg, corticosteroids, infliximab, adalimumab	Well on therapy	Skenteris et al. (16)
18	G5-4	M	24 days	p.V434L	Antagonist binding site	Germline inherited	NR	**Heme:** anemia **Imm:** NR **BM:** myeloid hypoplasia, granulocytic hypoplasia, erythroid hyperplasia **Other:** familial history of hematologic malignancies/autoimmunity	• NF-κB reporter assay • TLR8 stimulation–dependent NF-κB and cytokine production in THP-1 cells	Corticosteroids	Deceased, uncertain cause	Liang et al. (17)
19	Mother #11	F	NR	p.L433F	Antagonist binding site	Germline	50%	**Heme:** AIHA, prolonged B cell lymphopenia after rituximab **Imm:** low IgM/IgD, reduced total B cells **BM:** NR **Other:** family history of thrombocytopenia in mother	• Mother of #11	Corticosteroids, rituximab	Well	Barber et al. (18)

Ref ID, patient ID in respective publication; Age, age at which symptoms arose; F, female; M, male; NR, not reported; VAF, variant allele frequency; Heme, hematologic manifestations; Imm, immunophenotyping findings; BM, bone marrow; LGL; large granular lymphocytes; T-LGLL, T cell large granular lymphocytic leukemia; Ig, immunoglobulin; DNT, double-negative T CD4^−^CD8^−^ cells; Th1, T helper 1 (CD4^+^CXCR3^+^CCR6^+^) cells; AIHA, autoimmune hemolytic anemia; HLH, hemophagocytic lymphohistiocytosis; HSM, hepatosplenomegaly; LAD, lymphadenopathy; GI, gastrointestinal; pRBC, packed red blood cells; Validation, as reported in reference paper; NF-κB reporter assay, NF-κB reporter SEAP assay in HEK Null1 cells with ectopic expression of respective TLR8 variant; IVIg, intravenous immunoglobulin replacement therapy; PBMCs, peripheral blood mononuclear cells; JAKi, JAK inhibitor; MTX, methotrexate; HCT, hematopoietic cell transplant; s/p, status post; f/u, follow-up; Ref, respective reference publication.

**Figure 1.**
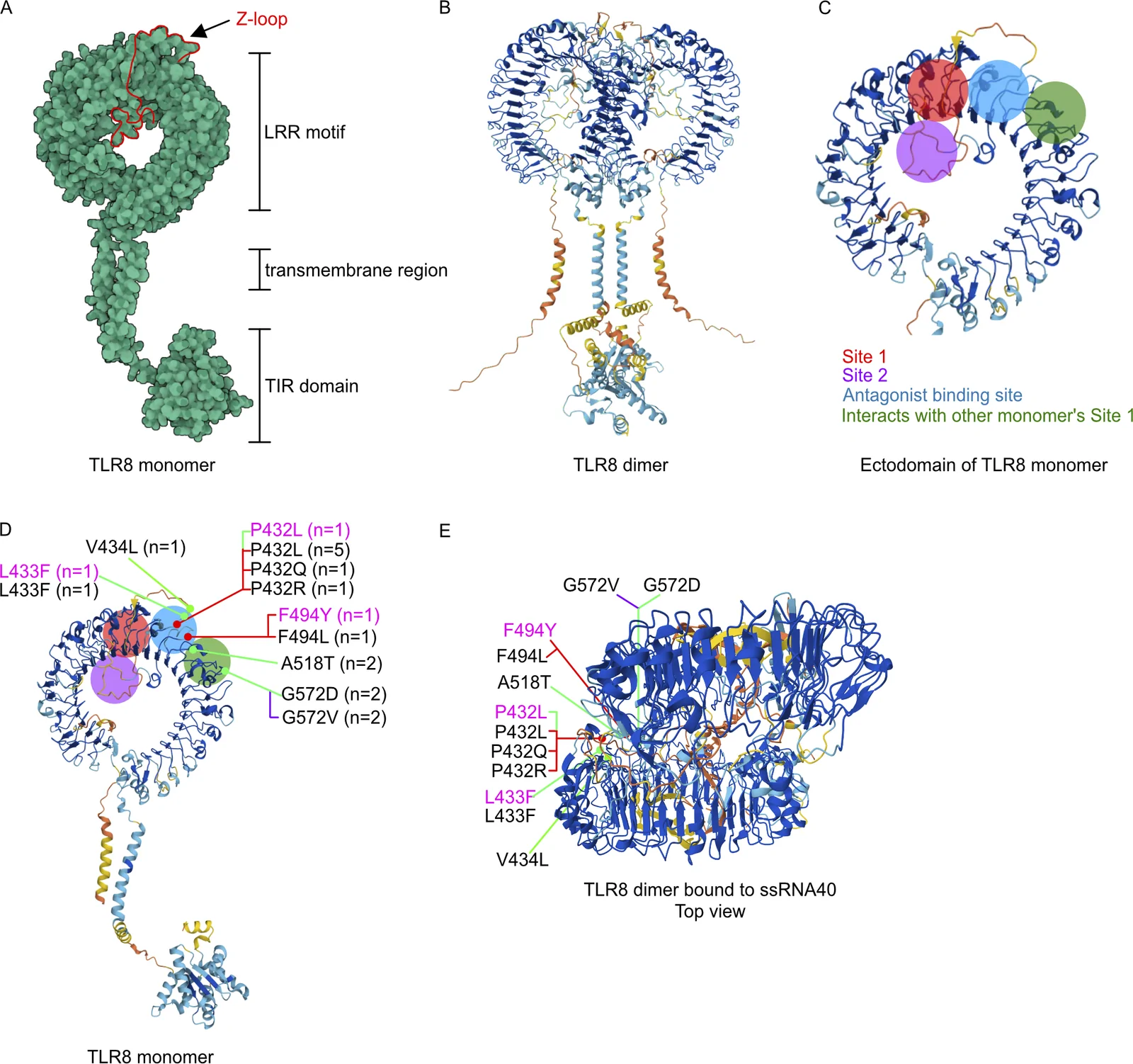
**Computational modeling of TLR8 monomer and homodimer reveals clustering of TLR8 GOF variants at sites of agonism and antagonism. (A)** TLR8 monomer represented as a space-fill model with domains notated. **(B)** TLR8 homodimer in inactive open conformation. **(C)** Site 1 (red, binds uridine), Site 2 (purple, binds short uridine-rich motifs), the antagonist binding site (blue, binds 3′-mer oligos), and a region that interacts with the other monomer’s Site 1 (green) are notated in the ectodomain of the TLR8 monomer. **(D and E)** Two perspectives of TLR8 GOF variants clustering at the dimer interface in (D) monomer. **(E)** Top view of the active dimer conformation with ssRNA40 bound (one per monomer, in yellow). Variants are notated only on the top monomer. (A–D) Models were derived from the AlphaFold Protein Structure Database (AF-Q9NR97-F1) (48), and the E model was derived from the RCSB Protein Data Bank (4R08) (49). Pink text: female; black text: male; purple line: reduced protein stability leading to molecular LOF with heightened response to TLR8 stimulation leading to phenotypic GOF; green line: germline; red line: mosaic.

TLR8 has historically been understudied relative to other TLRs, in part due to the murine ortholog partially lacking single-stranded RNA (ssRNA)–sensing capacity as the human protein (34, 35, 36, 37, 38). However, the discovery of GOF variants in *TLR8* leading to a monogenic IEI has transformed the understanding of TLR8 as a critical regulator of myeloid immune homeostasis (11). TLR8 is an endosomal receptor that is highly expressed in myeloid cells and largely absent in B or T lymphocytes (34, 39, 40, 41, 42, 43, 44). TLR8 functions as a homodimer and recognizes degradation fragments of ssRNA derived from pathogens including viruses, bacteria, and protozoa, as well as endogenous self-RNA (45). Structurally, each TLR8 monomer contains 26 LRRs that mediate ligand recognition with a z-loop inserted between LRR14 and LRR15 (residues 433–481) that must be cleaved for dimer formation (Fig. 1 A) (46). At baseline, TLR8 exists as a preformed homodimer in an inactive open conformation (Fig. 1 B) (47). Upon ligand binding, conformational changes bring the two C-terminal TIR domains closer in proximity, enabling downstream signaling through MyD88 (46). Here, we review current knowledge of the molecular, cellular, and clinical features of a spectrum of disease associated with TLR8 GOF and highlight unresolved questions important for understanding the mechanisms of disease.

## TLR8 activation is controlled by three distinct structural sites

TLR8 shares structural and mechanistic similarity to TLR7, also an endosomal DAMP and PAMP sensor that recognizes RNA degradation products (37, 50, 51, 52, 53), and the genes encoding TLR7 and TLR8 are adjacent to each other on the short arm of the X chromosome (54, 55, 56). Structurally, TLR7 and TLR8 each have three sites critical for binding agonists and antagonists (Fig. 1 C). Both share a conserved Site 1, which binds single nucleosides (TLR7: guanosine or 2′,3′-cGMP; TLR8: uridine) (57, 58). Site 2 differs in structure and motif preference; however, both TLR7 and TLR8 Site 2 regions bind to short uridine-rich motif (e.g., UUU, UG) oligonucleotides (46, 49, 59). Site 2 binding is auxiliary, enhancing binding affinity of Site 1 rather than being responsible for activation alone (59, 60). Recently, a third site was discovered as an antagonist binding region by the Gantier lab (61). They demonstrated that binding of 3-mer oligos of 2′-*O*-methyl (2′-OMe)-guanosine RNA with a 5′-guanosine within this site (TLR7: P435, F506, F507; TLR8: P432, F494, F495) stabilizes the inactive open dimer conformation of both proteins (27, 61). In mammalian cells, there are at least 31 ribosomal RNA (rRNA) sites that contain 2′-OMe guanosines (62); thus, this pool of fragmented rRNA may serve as a mechanism to dampen TLR7 and TLR8 signaling at baseline and prevent autoinflammation (61). Notably, binding at this antagonistic site prevents Site 1 and 2 binding, and vice versa (61). Thus, this finding established a competitive model in which agonists compete with antagonists bound to TLR7 and TLR8 at basal levels for control of the conformational state, subsequently switching signaling capacity of the receptors on and off (61). Thus, activation of both TLR7 and TLR8 likely depends upon the displacement of endogenous 2′-OMe guanosine from the antagonist binding site following substantial accumulation of agonistic RNA degradation products in the endosome, a protective mechanism allowing for sensing of pathogens while reducing autoinflammation.

Structural analyses have localized these three functional sites to distinct areas of the receptors’ ectodomains (46, 59, 60, 61, 63) (Fig. 1 C). In TLR8, Site 1 consists of the LRR11-14 region of one monomer and LRR16-18 of the other monomer, where conserved residues coordinate nucleoside binding and are essential for receptor dimerization (46, 47, 59). Site 2 is formed at the dimer interface in the LRR10-13 region, where short uridine-rich oligonucleotides make contacts with residues from both monomers simultaneously (46, 49, 64). The antagonist binding site is principally defined by amino acid residues F494 and F495 in LRR15-16, with P432 in the z-loop contributing indirectly to the geometry of the antagonist pocket (16, 61). Of significance, all known TLR8 GOF-causing variants cluster at residues important for or adjacent to Site 1 in the dimer (p.A518T, p.G572D, p.G572V) or the antagonist binding site (p.P432L, p.P432Q, p.P432R, p.L433F, p.V434L, p.F494Y, p.F494L, with additional evidence for interaction of p.A518T as well) (Fig. 1 and Table 1) (11, 12, 46, 49, 61).

## Disease associated with TLR8 GOF: Clinical phenotypes and genetics

To date, 19 patients with 10 variants in *TLR8* have been reported (Table 1) (11, 12, 13, 14, 15, 16, 17, 18). Proteins encoded by reported variants have been functionally characterized as GOF primarily through *in vitro* NF-κB reporter assays utilizing ectopic expression of *TLR8* variants, phosphorylated NF-κB in peripheral blood mononuclear cell samples or patient induced pluripotent stem cell (iPSC)–derived myeloid cells, and/or quantification of basal and TLR8 stimulation–dependent cytokine production in primary patient samples or cell lines expressing variants of interest.

### Initial discovery: INFLTR8 phenotype—neutropenia with lymphoproliferation and BM failure

We originally identified 6 male patients who had a severe inflammatory phenotype with lymphoproliferation, neutropenia and other cytopenias, recurrent infections, and in many cases BM failure, all of whom had novel mosaic or germline GOF variants in *TLR8* (11). Immunophenotyping identified expanded CD8^+^ T cells with a terminally differentiated effector memory T cell reexpressing CD45RA (TEMRA) phenotype, impaired B cell maturation and hypogammaglobulinemia with some patients having near-absent B cells, marrow infiltration by activated CD8^+^ T cells, and strongly elevated serum cytokines including IL-12, IL-18, and interferon (IFN)-γ, as well as soluble IL-2Rα (11). Most patients (5/6) had disease due to genetic mosaicism, with variant allele fractions (VAFs) between 8 and 26%. Of note, 4 patients had the same mosaic p.P432L variant. One patient who presented in infancy had a *de novo* germline variant (Table 1, #6). In 3 mosaic patients, VAFs were similar across hematopoietic lineages and between peripheral blood cells and skin fibroblasts, suggesting a postzygotic mutational event early in embryogenesis. One patient had mosaicism limited to the hematopoietic compartment, evidenced by the absence of the variant in a skin fibroblast line (11). The fact that variants with such low VAF could cause severe systemic disease leading to BM failure suggested that TLR8 GOF is a cell-extrinsic disease driven by a small fraction of cells.

Recently, we reported an additional 4 patients, for a total cohort of 10, and their treatment outcomes, including the first female with TLR8 GOF (with germline p.P432L) who had onset of disease at 2 wk of age (Table 1, #1–10) (12). 8 of the 10 patients had genetic variants at the same amino acid in the antagonist binding site, p.P432, with 3 of the 6 possible amino acid substitutions at that position identified in patients with TLR8 GOF thus far. Most recently, we identified a woman in her 70s with a 20-year history of refractory neutropenia, severe infection, CD8^+^ T cell infiltration in her BM, transient large granular lymphocytes (LGL) on a peripheral blood smear, and undetectable B cells with antibody deficiency associated (14). She has a mosaic variant at the amino acid position 494 in the antagonist binding site (p.F494Y, #13 in Table 1) leading to GOF, the same position as another patient from the original cohort (p.F494L, #3 in Table 1).

We have termed this severe spectrum of disease with neutropenia and BM involvement: inflammation, neutropenia, BM failure, and lymphoproliferation caused by TLR8 (INFLTR8) (11, 12).

### Growing spectrum of diseases associated with TLR8 GOF

**Figure 2.**
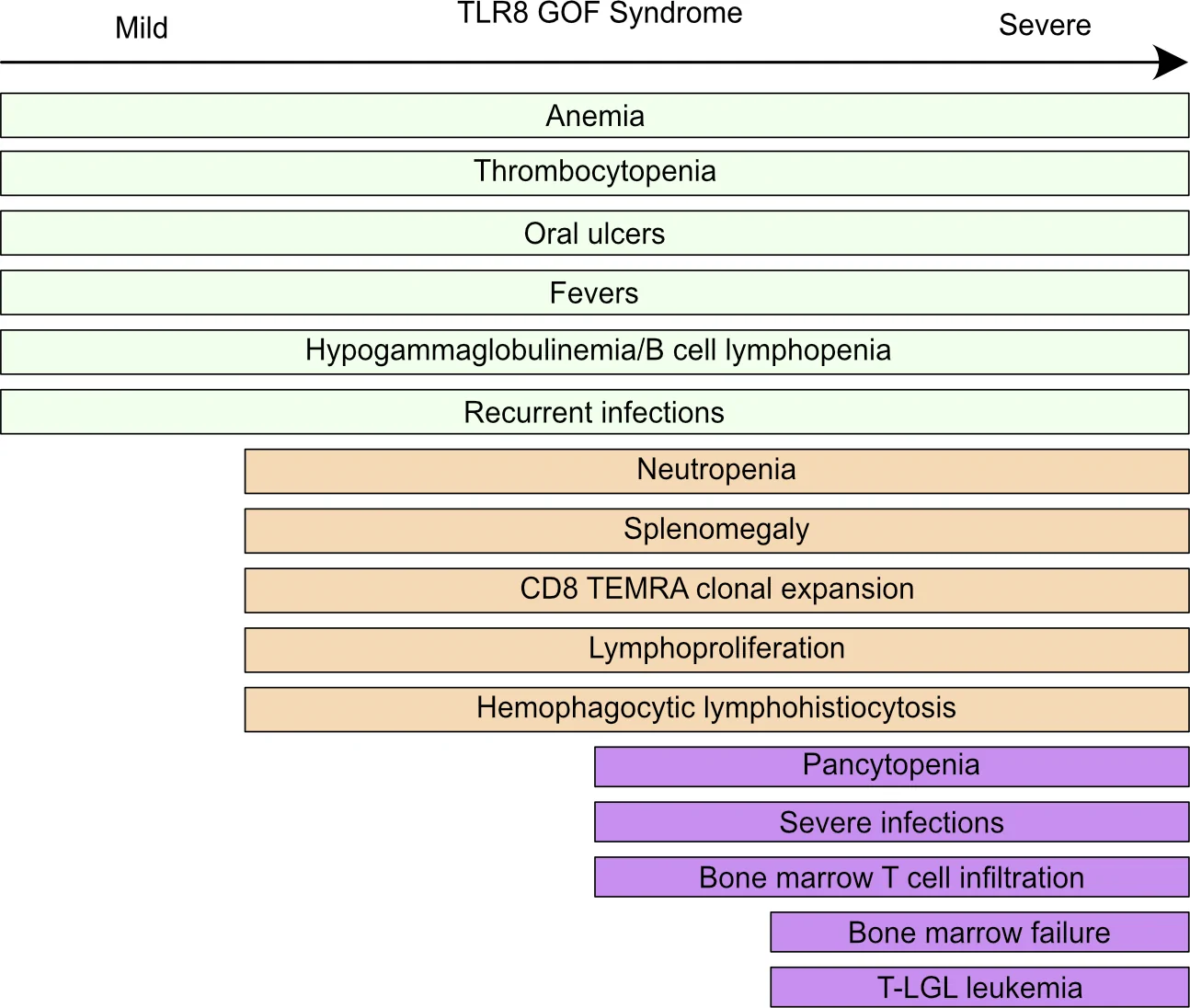
**Spectrum of TLR8 GOF phenotypes, separated in categories from mildest to most severe.** LGL, large granular lymphocytes; T-LGL leukemia, T cell LGL leukemia; TEMRA, terminally differentiated effector memory T cell re-expressing CD45RA.

Since the original discovery in 2021, a spectrum of TLR8 GOF has begun to emerge (Table 1, Fig. 2) (11, 12, 13, 14, 15, 16, 17, 18), with additional clinical phenotypes including hemophagocytic lymphohistiocytosis (HLH), autoimmunity, and relatively isolated anemia. Commonalities among most patients include cytopenias, infections, general symptoms of inflammation including fever and oral ulcers, and antibody deficiency with impaired B cell maturation (Fig. 2).

In general, severe disease is indicated by refractory neutropenia and BM involvement including infiltration of T cells, associated with significant mortality necessitating allogeneic hematopoietic cell transplant (HCT) in most patients. Consistent with this, Qu et al. identified another male patient with a germline *de novo* variant at a residue critical for interacting with Site 1 within the dimer, p.G572D, a variant previously identified and functionally validated in our original cohort (11, 13). The patient presented with infections, refractory neutropenia, and lymphoid hyperplasia with T cell infiltrate in the BM, anemia, and what was described as autoimmune myelofibrosis of the BM, similar to other patients with INFLTR8 phenotype.

Barber et al. expanded the phenotypic spectrum by identifying a young male with HLH associated with a germline variant in the antagonist binding site (p.L433F, #11 in Table 1) (18). While distinct from prior patients, he had refractory neutropenia and T cell infiltration on BM biopsy characteristic of other patients with INFLTR8. Hemophagocytosis, without meeting criteria for HLH, was observed in one other patient in the liver (Table 1, #1); however, this is the first report of an HLH phenotype due to TLR8 GOF. The mother of this patient (Table 1, #19) also carried the same variant and had a history of autoimmune hemolytic anemia (AIHA) and prolonged B cell lymphopenia after rituximab therapy (with the maternal grandmother of unknown genotype having thrombocytopenia). This suggests the possibility that in the germline state, this variant may have differing penetrance, potentially due to expression on the X chromosome.

### TLR8 GOF leading to anemia with variable inflammation and autoimmunity

Distinct from the INFLTR8 phenotype, Fejtkova et al. reported identical twins with AIHA, autoinflammation, and autoimmunity including arthritis, and central nervous system (CNS) vasculitis with a maternally inherited p.G572V variant (Table 1, #14 and #15) (15). Unlike in patients with INFLTR8, neither child was neutropenic, though they did have mildly reduced CD19^+^ B cell frequencies. Their mother, who carried the variant in the heterozygous state, had a history of autoimmunity with arthritis and antiphospholipid antibody syndrome without cytopenias. Both patients responded to immunosuppressive therapy, with one brother (Twin B) requiring HCT for severe CNS vasculitis. Despite the variant having heightened sensitivity to TLR8 stimulation in a cell line model, primary cells had increased degradation of the corresponding TLR8 protein (15). Further, the p.G572V variant increased both TLR8 and TLR7 signaling. Of interest, a full-body deletion of *Tlr8* in mice showed overactive Tlr7 in dendritic cells, suggesting that interactions between TLR7 and TLR8 regulate their activity (65). Thus, this unique disease phenotype in patients with the p.G572V variant with prominent autoimmunity may be driven predominantly by enhanced TLR7 activity rather than TLR8 hyperactivation. Indeed, these patients had increased levels of multiple type I IFNs more typical of TLR7 GOF than interferonopathies (15, 24, 66). This is in contrast to other patients with germline variants at the same amino acid position, p.G572D, who had more severe disease (Table 1), suggesting that there may be variable phenotypes associated with different missense variants at this amino acid position (11, 13).

Liang et al. reported a male infant presented at d24 of life with anemia with impaired erythropoiesis on BM biopsy, with a family history of hematologic malignancy and autoimmunity (Table 1, #18) (17). Whole-genome sequencing identified a p.V434L variant in the child and their unaffected mother, as well as a maternal grandmother who had pure red cell aplasia. The investigators suggested that there are intrinsic effects of TLR8 GOF on erythroid precursors leading to anemia in this case. The child’s anemia was improved with corticosteroids, but they subsequently died at a young age of unreported cause (17). Overall, this case suggests that some patients may have a more “mild” phenotype with relatively isolated anemia, although the early mortality and uncertain outcome of this patient make this challenging to know.

Finally, Skenteris et al. reported two male siblings with a maternally inherited p.A518T variant who presented with anemia, thrombocytopenia, and leukopenia without neutropenia; with recurrent infections, hypogammaglobulinemia, and splenomegaly in the first year of life (Table 1, #16–17) (16). Their mother was unaffected but had some mild changes in her B cell phenotype. The siblings were diagnosed with vasculitis, described as cerebral and polyarteritis nodosa, and received multiple therapies with remission for both brothers on anti-TNF therapy. Similar to the siblings with the p.G572V variant (15), they identified that the p.A518T variant led to lower abundance of the TLR8 protein with increased protein turnover, but increased NF-κB activation following stimulation of primary cells with a TLR8 agonist (16).

### Genetic mosaicism and TLR8 GOF

Half of identified TLR8 GOF patients have disease due to mosaic variants in *TLR8* with VAFs ranging from 7 to 26%, including one female (Table 1), (11, 12, 14). Mosaicism has been detected not only in peripheral blood but is also present at similar frequencies in skin-derived fibroblasts and saliva in some patients (11, 12). This suggests that somatic mutations in *TLR8* can arise early in embryonic development and that TLR8 GOF variants may not confer a selective survival advantage or disadvantage to cells of the hematopoietic compartment, including immune cells. Mosaicism additionally demonstrates that a small population of GOF cells exerts a dominant effect on the immune system disproportionate to their numerical frequency, indicating a cell-extrinsic rather than cell-intrinsic mechanism of disease.

### TLR8 GOF is an X-linked dominant IEI

While most patients with this X-linked disease are male, three female patients with TLR8 GOF have been identified, including one with early-onset germline disease, further demonstrating the dominant nature of TLR8 GOF (12, 14, 18). Interestingly, a prior study demonstrated that *TLR8* may escape X chromosome inactivation (XCI) in human monocytes, suggesting that in females both WT and GOF TLR8 protein may be co-expressed in the same myeloid cell (56). XCI studies in an affected infant female with a germline variant (Table 1, #10) showed no skewing (12), while an older woman with a mosaic variant (Table 1, #13) had significant skewing of 95% (14), although it is unknown whether that was due to the *TLR8* variant or age-acquired changes (67, 68). The final female patient (Table 1, #19) had a milder phenotype than her son who required HCT, and thus clinically may be characterized as having a phenotype of anemia with autoimmunity (18).

Of note, there are two other families with maternal transmission where females do not have a phenotype clearly related to TLR8 GOF (Table 1, #14–15, #16–17), both at positions important for Site 1 (p.G572V and p.A518T). One mother had no reported symptoms, while the other had rheumatologic diagnoses including arthritis and antiphospholipid antibody syndrome, though of uncertain relationship to her *TLR8* variant. In contrast, the three affected females all had variants at the antagonist binding site. This suggests that penetrance of the clinical phenotype in females may be related to the affected region.

It is uncertain whether the predominance of males identified thus far is due to a difference in expression of the GOF allele based on X chromosome dosage. One possible explanation is that the majority of patients identified had mosaic disease identified by exome. It is technically more challenging to identify a female with similar mosaicism by exome due to differences in VAF than a male; e.g., a male with 7% of cells harboring a GOF allele will have a VAF of 7%, while a female with the same 7% cells positive for GOF would have a VAF of 3.5% due to two X chromosomes per cell. The limit of detection of mosaic variants by exome is ∼10% depending on read depth; thus, approaches including deep targeted sequencing may be important for identifying patients, including females (69). It is also possible that a WT-GOF heterodimer formation could attenuate net GOF signaling output, leading to a milder phenotype in some females, though this has not been directly demonstrated in patient cells.

## Immunologic changes associated with TLR8 GOF

The TLR8 protein is expressed by myeloid cells including neutrophils, monocytes, macrophages, and myeloid dendritic cells, without significant expression in other cell types including adaptive lymphocytes (34, 39, 40, 42, 43, 44, 70). Despite this, immunophenotyping has demonstrated the most profound effects on T and B cells, indicating that the hyperinflammation in TLR8 GOF myeloid cells has far-reaching effects (11, 12, 16). Consistent with this, patients have elevated circulating proinflammatory cytokines produced by both myeloid cells and T cells, suggesting possible myeloid-derived paracrine mediators of lymphocyte reprogramming and hyperinflammation (Table 1). As additional patients are identified with varying phenotypes of clinical disease, it may be possible to correlate genotype with immunophenotype to help identify clinical course and potential treatment strategies.

### Adaptive immune cell phenotype in TLR8 GOF

The immunologic signature of TLR8 GOF is dominated by a chronically activated and terminally differentiated T cell compartment, defects in B cell maturation, and refractory neutropenia (11, 12, 13, 14, 15, 16, 18). Common findings in the peripheral blood include an inverted CD4:CD8 ratio, a shift in the CD45RA:CD45RO ratio of CD4^+^ T cells away from naive cells, expansion of TEMRAs, and increased double-negative T cells expressing TCRαβ and/or TCRγδ (11, 12, 14, 15, 16, 18). Additional immunophenotyping in one patient also identified an increased percentage of CD4^+^ T helper 1 cells and elevated PD-1 expression on both CD4^+^ and CD8^+^ memory T cells, consistent with chronic antigen- or cytokine-driven stimulation rather than a discrete clonal event (12).

Increased TEMRAs in TLR8 GOF may directly contribute to neutropenia. TEMRAs are characterized by a CD45RA^+^CCR7^−^CD27^−^CD28^−^ surface phenotype, high expression of cytotoxic granule proteins including granzyme A/B and perforin, and the ability to potently target cell killing (71, 72). Whether T cell cytotoxicity is a primary driver of neutropenia or a secondary consequence of the broader inflammatory environment remains unresolved.

B cell abnormalities, including near-absent B cells in some cases, and hypogammaglobulinemia, are seen in many patients (11, 12, 14, 18). B cell immunophenotyping has shown significantly reduced class-switched memory B cells relative to age-matched controls (11, 12, 14, 15, 16, 18). Additional immunophenotyping has identified reduced frequency of CD24^hi^CD38^hi^ transitional B cells and CCR6^+^ naive B cells, with reduced CD21 surface expression on naive B cells in some patients (11, 16). Two patients showed a prominent CD21^lo/neg^ population (11, 16), which is associated with autoimmunity, common variable immunodeficiency, splenomegaly, and cytopenias (73, 74, 75). The combination of reduced transitional B cells and reduced memory B cells implies a defect in the marrow and in peripheral maturation of lymphocytes.

For patients with BM abnormalities (Table 1), findings have been variable but frequently characterized by marked myeloid hypoplasia with lymphoid infiltrates composed of activated CD8^+^ T cells (11, 12, 13, 14, 18). In 6 cases, patient BM displayed increased numbers of LGL cells, with 2 patients diagnosed with CD8^+^ T cell LGL (T-LGL) leukemia at a relatively young age (#4 and 9 here) (11, 12, 14). In addition, some patients reported to have milder phenotypes (p.L433F, p.V434L, and p.A518T, Table 1) have had familial history of anemia and BM malignancies, suggesting there may be variable penetrance or other modifying factors of clinical disease (16, 17, 18).

### Cytokines in TLR8 GOF patients reflect myeloid and T cell activation

TLR8 signals through the transcription factors NF-κB and IRF5 (1, 76, 77, 78). These signaling transduction pathways can lead to proinflammatory cytokine production and type I IFN production, respectively (32, 77). Most patients with TLR8 GOF, however, have evidence of heightened NF-κB activity rather than an IRF5-driven IFN signature. In addition, as TLR8 is not significantly expressed in plasmacytoid dendritic cells (pDCs), the main producers of IFNs (40, 79, 80, 81), TLR8 GOF immunopathology may be due more to NF-κB–mediated inflammation in myeloid cells rather than pDC-driven IFN signatures. Interestingly, patients with germline variants reported as TLR8 GOF but with reduced levels of the TLR8 protein, p.G572V and p.A518T, had evidence of elevated type I IFNs or IFN-stimulated genes in the peripheral blood in addition to increased NF-κB phosphorylation upon stimulation of primary cells with TLR8 agonists or at baseline, suggesting there may be some different signaling mechanisms dominating (15, 16).

Serum cytokine profiles of patients with TLR8 GOF are generally consistent with excessive NF-κB–mediated signaling in monocytes and macrophages and provide a possible paracrine link between myeloid TLR8 hyperresponsiveness and lymphocyte phenotypes. Cytokines and inflammatory markers elevated in the serum of patients include IFN-γ, IL-1β, IL-2, soluble IL-2Rα, IL-6, IL-12, IL-18, soluble Fas ligand (sFasL), BAFF, and CXCL9 (11, 12, 14, 15, 16, 18). IL-12 and IL-18 are of particular mechanistic interest as they are potent myeloid-derived drivers of CD8^+^ T cell differentiation toward effector and terminally differentiated fates, with the potential to reprogram T cells independent of TCR engagement through cytokine-driven bystander activation (82, 83, 84, 85, 86). Further, IL-12 and IL-18 in combination stimulate CD8^+^ T cells to produce IFN-γ (81, 84), which is elevated in patients along with CXCL9, an IFN-γ–induced chemokine (87, 88), suggesting heightened T cell activation (89). Lastly, elevated BAFF likely reflects reduction in B cell maturation or survival (90).

### Myeloid cell dysfunction in TLR8 GOF

The expression of the TLR8 protein is almost exclusively limited to myeloid lineage cells. Immunophenotyping of monocytes from patients has in some cases demonstrated small increases in overall frequency in the peripheral blood, with variable changes in nonclassical CD14^lo^CD16^+^ monocytes, but without consistent phenotypic changes in monocytes by flow cytometry (11, 15, 16, 17).

Identification of patients with genetic mosaicism has led to an interesting question: how do 7% of cells harboring a *TLR8* GOF variant cause disease? While analysis of peripheral blood monocytes has not identified enrichment of the variant in these cells in cases of mosaicism (11), one possibility is that GOF myeloid progenitors are selectively expanded in a tissue-specific inflammatory environment they themselves help create; chronic NF-κB activation promotes myeloid cell survival and proliferation, potentially increasing the effective GOF fraction over time. Testing this will require careful analysis of myeloid cell populations at sites of inflammation over time, e.g., BM and lymphoid organs, rather than circulating cells. Another possibility is that myeloid cells occupy strategic environments, particularly in the BM, where even a small number of hyperactivated cells can disproportionately shape the local cytokine milieu encountered by other developing hematopoietic progenitors and trafficking lymphocytes.

### Potential mechanisms of neutropenia in TLR8 GOF

Neutropenia is a hallmark of TLR8 GOF with BM involvement/INFLTR8 phenotype, but the mechanism(s) responsible remain unknown. Some clues may come from similarities to findings in patients with T-LGL leukemia. Overlapping findings between TLR8 GOF and T-LGL leukemia include expanded CD8^+^ T cells with a terminally differentiated phenotype (clonal in T-LGL leukemia and oligoclonal or nonclonal in TLR8 GOF), elevated cytokines including IFN-γ, which is associated with immune-mediated BM failure, prominent neutropenia, and in some patients pure red cell aplasia (91, 92, 93). In studies of T-LGL leukemia, proposed mechanisms of neutropenia include direct cytotoxic effects of the T-LGL cells on neutrophils, elevated circulating levels of sFasL, and autoimmune neutropenia (94, 95, 96), suggesting potential pathogenic mechanisms in TLR8 GOF.

**Figure 3.**
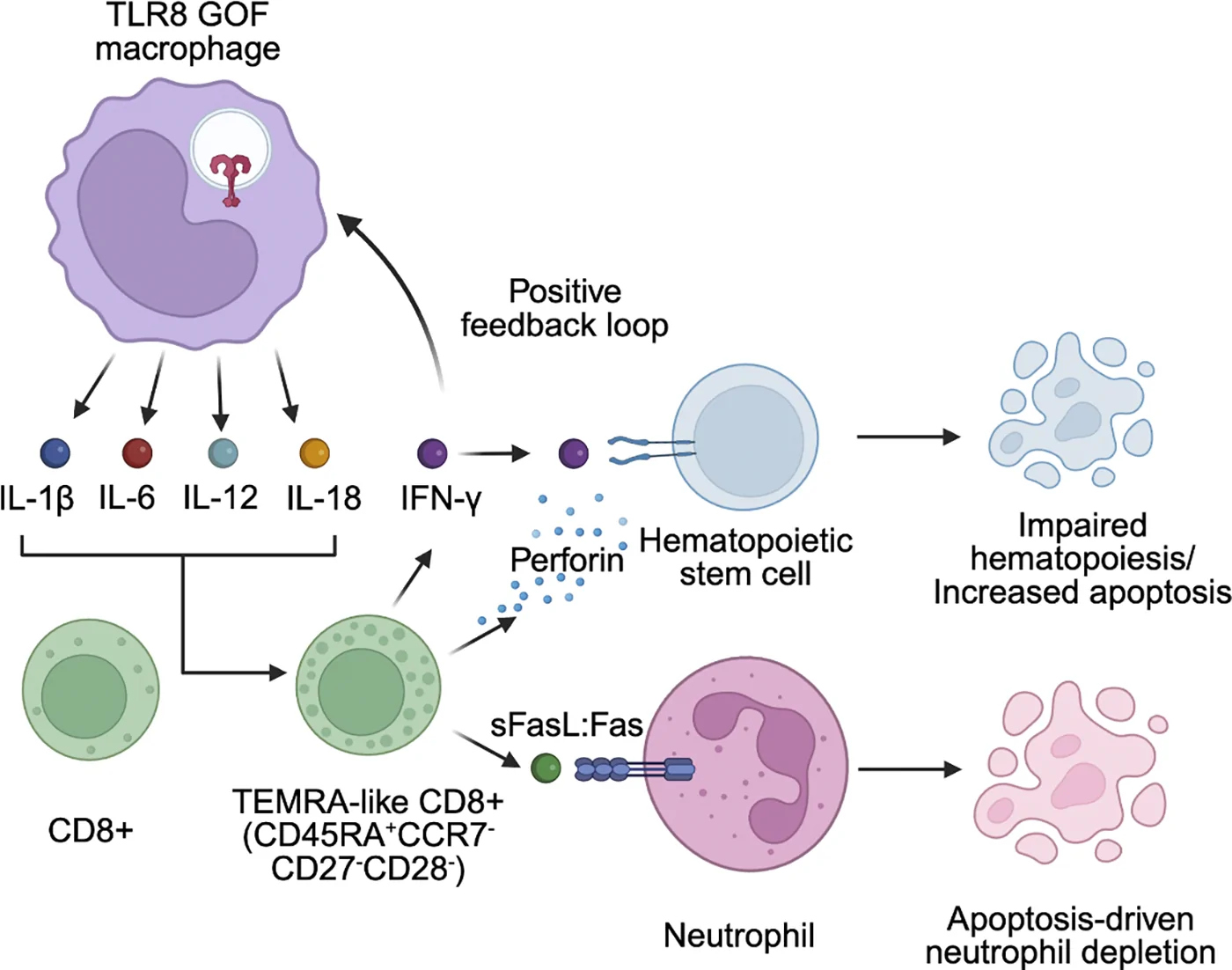
**Hypothesized model of immune dysregulation due to TLR8 GOF macrophages.** Macrophages expressing *TLR8* GOF variants produce hyperinflammatory cytokines. Patients have elevated myeloid and cytotoxic T cell–derived cytokines in their periphery, suggesting that cross-talk between TLR8 GOF macrophages (or other myeloid cells) and CD8^+^ T cells leads to reprogramming of CD8^+^ T cells to a TEMRA-like phenotype. This positive feedback loop could then lead to a hyperinflammatory environment and neutrophil apoptosis and BM failure driven by cytokines, including possibly Fas ligand (sFasL), in addition to potential direct cytotoxic effects of CD8^+^ T cells.

The adaptive immune profiles, cytokines, and myeloid hyperresponsiveness in TLR8 GOF combined with similarities to T-LGL leukemia suggest potential models of neutropenia due to heightened TLR8 signaling through NF-κB in myeloid cells, production of proinflammatory cytokines leading to CD8^+^ T cell activation, and a positive feedback loop. We hypothesize that neutropenia in patients is due to depletion of neutrophils through Fas ligand–mediated apoptosis, direct cytotoxic effects of terminally differentiated CD8^+^ T cells on hematopoietic progenitors or neutrophils, and/or effects of the hyperinflammatory environment (e.g., IFN-γ) (Fig. 3).

## Genetic and functional testing for TLR8 GOF

While detecting germline variants is now relatively standard, somatic variants with low VAF may escape detection by standard clinical testing; conventional sequencing techniques including exome sequencing typically have read depths of 100–200×, while genome sequencing has much lower coverage of ∼20–30× at any particular nucleotide (97). Thus, identifying mosaicism requires careful review of sequencing data or alternative approaches including targeted deep sequencing, with several clinically available deep-sequencing tests becoming more widely available (69). Validation of the findings using an orthogonal approach such as droplet digital PCR from multiple tissue samples may also be required to confirm sequencing findings.

Once a potentially pathogenic variant is identified, functional testing to confirm its pathogenicity is necessary to begin delineating disease mechanisms. Most variants have undergone functional testing using an *in vitro* protocol in which ectopic expression of *TLR8* variants in a cell line lacking endogenous TLR8 and expressing an NF-κB reporter (TLR8-null HEK-Blue Null1 cells) was used. By stimulating these cells with increasing doses of TLR8-specific agonists, such as TL8-506, activity can be compared with controls (11, 12). The GOF phenotype may not be obvious at lower or higher doses; thus, it is important to test a range of agonists. It is also possible that such testing may reveal a strength of signaling that correlates with disease. For example, we demonstrated that the p.V434L variant, associated with relatively isolated anemia, was only GOF with strong stimulation as compared to other variants associated with the more severe INFLTR8 phenotype (12).

Testing of primary patient cells is important for determining functional effects of TLR8 GOF in a relevant cell type. In patients with an INFLTR8 phenotype with mosaicism, functional studies demonstrated that a small percentage of monocytes (5–6%) had heightened phosphorylation of NF-κB in response to a low dose of TLR8 agonist, suggesting effects due to the presence of the variant in a subpopulation of cells (11). Disease due to genetic mosaicism presents challenges in assays of primary cells as it is not possible to delineate cells expressing WT versus GOF *TLR8*. To overcome this limitation, we utilized iPSCs derived from patients with mosaicism and differentiated cells into macrophages or neutrophils expressing either WT or GOF variant *TLR8*, thereby offering a mechanism to directly test the effects of variation in *TLR8* only. In this model, cells demonstrated hyperphosphorylation of NF-κB upon stimulation with a TLR8 ligand, with corresponding elevated cytokine production (11, 12). The major advantage of this system is the ability to generate isogenic pairs—WT and GOF iPSC lines from the same individual—enabling mechanistic comparisons unconfounded by interindividual genetic variation.

Distinct from other variants, TLR8 GOF due to p.A518T and p.G572V associated with a phenotype of anemia and autoimmunity (Table 1) displayed GOF activity in primary cells and cell line systems, but at the same time led to reduced levels of the TLR8 protein (15, 16). Stimulation of primary monocytes from patients with both variants with TLR8 ligands demonstrated increased production of inflammatory cytokines compared with healthy controls (15, 16). Interestingly, stimulation with a TLR7 ligand in a patient with a p.G572V variant also showed heightened response to the TLR7-specific agonist imiquimod, suggesting an imbalance of TLR7/TLR8 signaling (15). The expression of variants in HEK cells followed by treatment with an inhibitor of new protein synthesis showed that both p.A518T and p.G572V protein variants degraded faster compared with WT (15, 16). Skenteris et al. further compared both variants and showed increased proteasomal degradation of the p.A518T, but not p.G572V, variant, as partially responsible for protein loss (16).

Together, testing of identified variants thus far suggests that demonstrating hyperresponsiveness to TLR8 signaling through cell line models, primary cells, and/or iPSC-derived myeloid cells can identify TLR8 GOF through measurement of NF-κB activity and/or cytokine production. At the same time, additional nuances including alterations of TLR8 protein stability and possibly TLR7 signaling may modify signaling pathways, and it will be interesting to determine how they may affect the clinical phenotypes of patients and potential treatment options.

## Treatment for TLR8 GOF

There are no approved targeted therapies for TLR8 GOF. In the most severe cases, patients have tended to have an overall poor response with immunosuppressive treatment. Therapies that have not produced consistent clinical benefit in patients with INFLTR8 and neutropenia with BM involvement include G/GM-CSF, corticosteroids, azathioprine, thalidomide, mycophenolate mofetil, sirolimus, ruxolitinib, and rituximab (11, 12, 13, 17). Notably, neutrophil counts are refractory to nearly all therapies and neutropenia is a major driver of morbidity in INFLTR8 due to overwhelming opportunistic infections. Of the 13 patients with INFLTR8 phenotype, 4 have died from infection or BM failure (11, 12). Allogeneic HCT represents the only treatment demonstrated to produce durable remission, with six patients benefiting from transplantation. However, 2 of the 8 transplanted patients died from transplant-related complications, reflecting the severity of preexisting disease at the time of conditioning (12).

### Supportive care

Most patients with TLR8 GOF have significant infection risk. Supportive care is a critical component of TLR8 GOF management given the combined immunodeficiency driven by neutropenia and hypogammaglobulinemia. IVIg replacement therapy is used to reduce infectious burden in patients with low immunoglobulin levels, and has been administered alongside other therapies (11, 12, 14). Prophylactic antimicrobial therapy is similarly important given the high rates of serious bacterial and fungal infections seen across patients (11, 12). Although growth factors (G-CSF and GM-CSF) have been used in attempts of improving neutrophil counts, it has not produced consistent or durable benefit in most patients, with the notable exception of an elderly female with mosaic disease who has stabilized with growth factor with prednisone and a JAK inhibitor (11, 12, 14).

### Hematopoietic cell transplant

Allogeneic HCT is currently the only intervention demonstrated to produce durable disease remission in patients with INFLTR8 phenotype (neutropenia and BM involvement) and has led to sustained correction of cytopenia and immune dysregulation in 5 patients (12). The progressive nature of BM failure in INFLTR8 argues for early referral before irreversible hematopoietic damage or infectious burden accumulates. While the optimal approach to HCT is uncertain, complications in patients thus far have included high rates of cytopenias after transplant and graft-versus host disease. The mosaic nature of the disease suggests that 100% donor chimerism is required for remission, particularly in the myeloid compartment, with the caveat that it is possible that residual tissue-resident myeloid cells expressing *TLR8* GOF may contribute to posttransplant complications despite the appearance of complete chimerism in the periphery. In addition, our experience suggests avoiding high-dose busulfan due to concern for therapy-related toxicity (12). A final consideration is to use agents to reduce inflammation prior to transplant, including JAK inhibitors used in 2 patients and emapalumab (anti-IFN-γ monoclonal antibody) in another patient (12, 18).

### Cytokine blockade and JAK inhibition

The finding that hyperinflammatory cytokines are consistently elevated in patients with TLR8 GOF provides a strong rationale for cytokine-directed therapy. Two brothers with cytopenias, infections, and vasculitis associated with a p.A518T variant had a positive response to anti-TNF therapy in combination with IVIg (16). This suggests that in some patients, blocking a single cytokine can produce meaningful clinical benefit possibly related to severity of disease and clinical symptoms (16).

As noted above, emapalumab was successfully used in a patient with an HLH phenotype due to a germline p.L433F *TLR8* variant (Table 1, #11) as a “bridge” to successful allogeneic HCT, reflecting the rationale for targeting elevated IFN-γ levels seen across TLR8 GOF patients (18). JAK inhibitors, which broadly suppress cytokine signaling downstream of multiple receptors, have been used in a subset of patients without consistent benefit (12), though this may reflect insufficient drug exposure, late initiation, or irreversibility of BM damage at the time of treatment. However, such agents may have an important role in the pretransplant phase to reduce inflammation prior to HCT. Of note, the elderly female we recently reported with a p.F494Y variant has demonstrated sustained clinical benefit from a combination that included JAK inhibition, suggesting that this approach may be effective in some patients, particularly those who are not transplant candidates (14).

### Other potential targeted therapies

CU-CPT9a is a small TLR8-specific antagonist that binds to the inactive TLR8 dimer (98). It has been shown to potently suppress WT TLR8 signaling but fails to inhibit p.P432L TLR8, the most common GOF variant (12). However, we did observe a reduction in inflammatory cytokine production in iPSC-derived macrophages harboring the p.P432L variant following TLR8 stimulation when pretreated with chloroquine, a drug that inhibits endosomal TLR signaling, or CPG-52364, a small-molecule inhibitor of TLR7/8 (12). These findings suggest that there may be mechanisms to inhibit TLR8 signaling in patients, although there was unclear benefit of hydroxychloroquine treatment in two patients (Table 1) (12, 15).

Resistance to antagonism associated with many of the variants has significant therapeutic implications, which may mean that direct TLR8 antagonism will require variant-specific or alternative approaches. Oligonucleotide antagonists, such as 2′-OMe-guanosine RNA fragments that bind to the antagonist binding site, additionally highlight different variant resistance profiles. The Gantier lab demonstrated that the p.G572D TLR8 GOF variant was able to be suppressed with 2′-OMe–modified guanosine RNA fragments, yet the p.F494L variant was not, indicating again that the resistance landscape varies by variant, target site, and the antagonist (61). Several small-molecule dual TLR7/8 antagonists, for example, E6742, enpatoran, and afimetoran, are undergoing clinical trials for inflammatory disease and may soon reveal more immediate translational paths for TLR8 GOF (99, 100, 101). Variants that remain sensitive to antagonism, such as those affecting p.G572, may be amenable to this approach.

TLR8 signaling utilizes the MyD88-IRAK4 Myddosome (1, 76, 102). Inhibiting IRAK4 or other Myddosome-associated signal transducers therefore represents a therapeutic strategy that bypasses the problem of variant-specific antagonist resistance. Several IRAK4 inhibitors are currently in early clinical trials for inflammatory conditions including rheumatoid arthritis and myeloid malignancies, and preclinical data support their efficacy in suppressing TLR7/8-dependent and NF-κB–mediated cytokine production (103, 104, 105). Supporting this approach, IRAK4 inhibition was shown to suppress cytokine production *in vitro* in cells carrying activating UNC93B1 variants that exacerbate TLR7/8 activity (106). Their utility in TLR8 GOF has not yet been tested and may have significant off-target effects given that it would likely be required long term.

## Conclusions

The identification of TLR8 GOF disease has repositioned a long-neglected receptor as an important mediator of myeloid immune function. INFLTR8 illustrates how a defect confined to the innate compartment, often to a minor mosaic population of myeloid cells, can remodel the entire immune landscape to drive CD8^+^ T cell reprogramming, neutropenia, and, at its most severe, BM failure. In the spectrum of TLR8-associated disease, the recurring theme across the molecular, cellular, and clinical evidence is that TLR8 GOF is fundamentally a cell-extrinsic, cytokine-driven disease in which a small number of hyperactivated myeloid cells dictate the phenotype and functional capacity of immune subsets that do not themselves express TLR8.

Identifying therapies for affected patients will require further mechanistic understanding, including clarifying how myeloid hyperactivation leads to shifts in immune cell frequency and phenotype that define TLR8 GOF and INFLTR8. Specifically, discerning the key inflammatory drivers leading to CD8^+^ T cell dysfunction, mechanisms of neutropenia and B cell lymphopenia, and triggers of TLR8 activation including significance of variants in the antagonist binding site will be important. Such questions will require model systems that capture the disease’s defining feature: the interaction between a population of GOF myeloid cells and the adaptive immune cells they reprogram. There are direct therapeutic implications, including identifying patients who should proceed to early transplant and who may benefit from “bridging” therapies including anticytokine agents.

Additional discovery of genotype-, site-, and potentially sex-specific effects of *TLR8* GOF variants may help to predict disease severity and guide early intervention. A common polymorphism in *TLR8*, rs3764880 (p.Met1Val), leads to a reduction in the primary *TLR8* transcript and a shift toward a longer isoform, with studies suggesting reduced inflammation (107, 108). Given that the minor allele frequency of this variant ranges from ∼20 to 80% (109, 110), it will be interesting to determine whether this polymorphism or other cis-regulatory elements modify the severity or penetrance of TLR8 GOF. The heterogeneity of TLR8 GOF variants’ response to antagonism and patients’ refractoriness against some therapies argues for variant- or patient-specific therapy matched to mechanism rather than a single standardized approach.

Finally, TLR8 GOF serves as a natural model for understanding innate immune dysregulation by which localized myeloid hyperactivation reshapes systemic immunity. As one of the growing number of genetic errors of immunity caused by a single innate sensor, TLR8 GOF offers a valuable framework to inform the broader field of TLR-driven inflammatory disease.

## References

[1] Kawai, T., and S. Akira. 2010. The role of pattern-recognition receptors in innate immunity: Update on toll-like receptors. Nat. Immunol. 11:373–384. 10.1038/ni.1863 20404851

[2] Kawai, T., M. Ikegawa, D. Ori, and S. Akira. 2024. Decoding toll-like receptors: Recent insights and perspectives in innate immunity. Immunity. 57:649–673. 10.1016/j.immuni.2024.03.004 38599164

[3] Poli, M.C., I. Aksentijevich, A.A. Bousfiha, C. Cunningham-Rundles, S. Hambleton, C. Klein, T. Morio, C. Picard, A. Puel, N. Rezaei, . 2025. Human inborn errors of immunity: 2024 update on the classification from the International Union of Immunological Societies Expert Committee. J. Hum. Immun. 1:e20250003. 10.70962/jhi.20250003 PMC12829761 41608114

[4] Bousfiha, A.A., L. Jeddane, A. Moundir, M.C. Poli, I. Aksentijevich, C. Cunningham-Rundles, S. Hambleton, C. Klein, T. Morio, C. Picard, . 2025. The 2024 update of IUIS phenotypic classification of human inborn errors of immunity. J. Hum. Immun. 1:e20250002. 10.70962/jhi.20250002 PMC12829316 41608113

[5] Zhang, S.-Y., E. Jouanguy, S. Ugolini, A. Smahi, G. Elain, P. Romero, D. Segal, V. Sancho-Shimizu, L. Lorenzo, A. Puel, . 2007. TLR3 deficiency in patients with herpes simplex encephalitis. Science. 317:1522–1527. 10.1126/science.1139522 17872438

[6] Lim, H.K., S.X.L. Huang, J. Chen, G. Kerner, O. Gilliaux, P. Bastard, K. Dobbs, N. Hernandez, N. Goudin, M.L. Hasek, . 2019. Severe influenza pneumonitis in children with inherited TLR3 deficiency. J. Exp. Med. 216:2038–2056. 10.1084/jem.20181621 PMC6719423 31217193

[7] van der Made, C.I., A. Simons, J. Schuurs-Hoeijmakers, G. van den Heuvel, T. Mantere, S. Kersten, R.C. van Deuren, M. Steehouwer, S.V. van Reijmersdal, M. Jaeger, . 2020. Presence of genetic variants among young men with severe COVID-19. JAMA. 324:663–673. 10.1001/jama.2020.13719 PMC7382021 32706371

[8] Zhang, Q., P. Bastard, Z. Liu, J. Le Pen, M. Moncada-Velez, J. Chen, M. Ogishi, I.K.D. Sabli, S. Hodeib, C. Korol, . 2020. Inborn errors of type I IFN immunity in patients with life-threatening COVID-19. Science. 370:eabd4570. 10.1126/science.abd4570 PMC7857407 32972995

[9] Asano, T., B. Boisson, F. Onodi, D. Matuozzo, M. Moncada-Velez, M.R.L. Maglorius Renkilaraj, P. Zhang, L. Meertens, A. Bolze, M. Materna, . 2021. X-linked recessive TLR7 deficiency in ∼1% of men under 60 years old with life-threatening COVID-19. Sci. Immunol. 6:eabl4348. 10.1126/sciimmunol.abl4348 PMC8532080 34413140

[10] Capitani, M., A.A. Al-Shaibi, S. Pandey, L. Gartner, H. Taylor, S.Z. Hubrack, N. Agrebi, M.J. Al-Mohannadi, S. Al Kaabi, T. Vogl, . 2023. Biallelic TLR4 deficiency in humans. J. Allergy Clin. Immunol. 151:783–790.e5. 10.1016/j.jaci.2022.08.030 36462956

[11] Aluri, J., A. Bach, S. Kaviany, L. Chiquetto Paracatu, M. Kitcharoensakkul, M.A. Walkiewicz, C.D. Putnam, M. Shinawi, N. Saucier, E.M. Rizzi, . 2021. Immunodeficiency and bone marrow failure with mosaic and germline TLR8 gain of function. Blood. 137:2450–2462. 10.1182/blood.2020009620 PMC8109013 33512449

[12] Arnold, D.E., S. Kaviany, J. Aluri, K.R. Calvo, S.S. Mehta, J.M. Tobin, L.G. Schuettpelz, N. Saucier, E.G. Schmitz, L. Murguía-Favela, . 2026. Clinical characteristics, management, and hematopoietic cell transplantation of patients with TLR8 gain-of-function. Blood Advances 10:1967–1976. 10.1182/bloodadvances.2025016338 PMC12997320 41370196

[13] Qu, S., P. Zhang, Z. Xu, T. Qin, B. Li, and Z. Xiao. 2026. Autoimmune myelofibrosis with a TLR8 gain-of-function defect. Leuk. Res. Rep. 25:100587. 10.1016/j.lrr.2026.100587 PMC13098463 42023410

[14] Patel, J.A., N. Saucier, H.M. Gil, E.G. Schmitz, M.B. Ruzinova, A. Saffaf, M. Blinder, N. Mehta-Shah, Z.D. Crees, M.A. Cooper, and O. Zimmerman. 2026. Mosaic TLR8 gain-of-function in an elderly female treated with JAK inhibitor and adjunctive therapy. J. Hum. Immun. 2:e20260113. 10.70962/jhi.20260113 PMC13603065 42782216

[15] Fejtkova, M., M. Sukova, K. Hlozkova, K. Skvarova Kramarzova, M. Rackova, D. Jakubec, M. Bakardjieva, M. Bloomfield, A. Klocperk, Z. Parackova, . 2022. TLR8/TLR7 dysregulation due to a novel TLR8 mutation causes severe autoimmune hemolytic anemia and autoinflammation in identical twins. Am. J. Hematol. 97:338–351. 10.1002/ajh.26452 34981838

[16] Skenteris, N.T., E. Luttermann, S. Nair, I. Evangelakos, M. Pujantell, M. Eggers, F. Hausmann, M. Bérouti, B. Padoan, F.J. Flomm, . 2026. Structural modeling and functional characterization of a novel gain-of-function TLR8 variant causing severe inflammatory syndrome. JCI Insight. 11:e187422. 10.1172/jci.insight.187422 PMC12956005 41729082

[17] Liang, J., Y. Wan, J. Gao, L. Zheng, J. Wang, P. Wu, Y. Li, B. Wang, D. Wang, Y. Ma, . 2024. Erythroid-intrinsic activation of TLR8 impairs erythropoiesis in inherited anemia. Nat. Commun. 15:5678. 10.1038/s41467-024-50066-w PMC11227506 38971858

[18] Barber, J.S., N. Saucier, M. Murphy, E.G. Schmitz, K. McDaniels, C. Cook, G. Venturi, J.W. Sleasman, K.M. Mahadeo, M.A. Cooper, . 2026. Familial TLR8 Gain-of-Function Presenting as HLH with Response to IFN-γ Blockade. J. Hum. Immun. e20260101. In press. 10.70962/jhi.20260101

[19] Brown, G.J., P.F. Cañete, H. Wang, A. Medhavy, J. Bones, J.A. Roco, Y. He, Y. Qin, J. Cappello, J.I. Ellyard, . 2022. TLR7 gain-of-function genetic variation causes human lupus. Nature. 605:349–356. 10.1038/s41586-022-04642-z PMC9095492 35477763

[20] David, C., M. Badonyi, R. Kechiche, A. Insalaco, M. Zecca, F. De Benedetti, S. Orcesi, L. Chiapparini, P. Comoli, S. Federici, . 2024. Interface gain-of-function mutations in TLR7 cause systemic and neuro-inflammatory disease. J. Clin. Immunol. 44:60. 10.1007/s10875-024-01660-6 PMC10850255 38324161

[21] Menon, S., D. Maurice, L.A. Robinson, J. Milner, V. Pascual, C.G. Vinuesa, and S. Kaicker. 2024. Refractory autoimmune thrombocytopenia in an infant with a de novo TLR7 gain-of-function variant. J. Clin. Immunol. 45:27. 10.1007/s10875-024-01824-4 PMC7616824 39433707

[22] Stremenova Spegarova, J.S., P. Sinnappurajar, D. Al Julandani, R. Navickas, H. Griffin, M. Ahuja, A. Grainger, K. Livingstone, G.I. Rice, F. Sutherland, . 2024. A de novo TLR7 gain-of-function mutation causing severe monogenic lupus in an infant. J. Clin. Invest. 134:e179193. 10.1172/jci179193 PMC11213501 38753439

[23] Zeng, Y., P. Tao, J. Wang, T. Li, Y. Du, X. Wang, W. Wang, S. Peng, W. Wang, M. Ma, . 2025. Somatic gain-of-function mutation in *TLR7* causes early-onset systemic lupus erythematosus. Ann. Rheum. Dis. 84:442–450. 10.1016/j.ard.2025.01.011 39919974

[24] Tusseau, M., C. Desvignes, G. Boursier, C. Fort, C. David, M.-L. Frémond, S. Benezech, S. Khaldi-Plassart, A. Chenel, J. Cognard, . 2026. Novel TLR7 gain-of-function variant and review of the associated disease spectrum. J. Hum. Immun. 2:e20250199. 10.70962/jhi.20250199 PMC12991832 41846847

[25] Parvaneh, N., H. Karami, L. Shahbaznejad, Z. Rezaei, P. Rostami, M. Shahrooei, and V. Ziaee. 2026. TLR7 F507S gain-of-function mutation presenting with early-onset SLE and hypertriglyceridemia: Response to JAK inhibition. J. Hum. Immun. 2:e20250196. 10.70962/jhi.20250196 PMC13177509 42170322

[26] Sethumadhavan, A., C. Mariasoosai, N. Yamakawa, N. Chamberlain, M.A. Deardorff, S. Funaki, T. Asano, J.-L. Casanova, H. Torabifard, B. Boisson, and E. Meffre. 2026. Identification of a novel TLR7 gain-of-function variant that underlies systemic lupus erythematosus. J. Hum. Immun. 2:e20250194. 10.70962/jhi.20250194 PMC12931374 41743833

[27] Crow, Y.J., and M.P. Gantier. 2026. TLR7 mutations leading to enhanced TLR7 signaling in humans. J. Hum. Immun. 2:e20250266. 10.70962/jhi.20250266 PMC7618847 41816251

[28] Döffinger, R., A. Smahi, C. Bessia, F. Geissmann, J. Feinberg, A. Durandy, C. Bodemer, S. Kenwrick, S. Dupuis-Girod, S. Blanche, . 2001. X-linked anhidrotic ectodermal dysplasia with immunodeficiency is caused by impaired NF-kappaB signaling. Nat. Genet. 27:277–285. 10.1038/85837 11242109

[29] Picard, C., A. Puel, M. Bonnet, C.-L. Ku, J. Bustamante, K. Yang, C. Soudais, S. Dupuis, J. Feinberg, C. Fieschi, . 2003. Pyogenic bacterial infections in humans with IRAK-4 deficiency. Science. 299:2076–2079. 10.1126/science.1081902 12637671

[30] Courtois, G., A. Smahi, J. Reichenbach, R. Döffinger, C. Cancrini, M. Bonnet, A. Puel, C. Chable-Bessia, S. Yamaoka, J. Feinberg, . 2003. A hypermorphic IkappaBalpha mutation is associated with autosomal dominant anhidrotic ectodermal dysplasia and T cell immunodeficiency. J. Clin. Invest. 112:1108–1115. 10.1172/JCI18714 PMC198529 14523047

[31] von Bernuth, H., C. Picard, Z. Jin, R. Pankla, H. Xiao, C.-L. Ku, M. Chrabieh, I.B. Mustapha, P. Ghandil, Y. Camcioglu, . 2008. Pyogenic bacterial infections in humans with MyD88 deficiency. Science. 321:691–696. 10.1126/science.1158298 PMC2688396 18669862

[32] Casanova, J.-L., L. Abel, and L. Quintana-Murci. 2011. Human TLRs and IL-1Rs in host defense: Natural insights from evolutionary, epidemiological, and clinical genetics. Annu. Rev. Immunol. 29:447–491. 10.1146/annurev-immunol-030409-101335 21219179

[33] Rael, V.E., J.A. Yano, J.P. Huizar, L.C. Slayden, M.A. Weiss, E.A. Turcotte, J.M. Terry, W. Zuo, I. Thiffault, T. Pastinen, . 2024. Large-scale mutational analysis identifies UNC93B1 variants that drive TLR-mediated autoimmunity in mice and humans. J. Exp. Med. 221:e20232005. 10.1084/jem.20232005 PMC11116816 38780621

[34] Jurk, M., F. Heil, J. Vollmer, C. Schetter, A.M. Krieg, H. Wagner, G. Lipford, and S. Bauer. 2002. Human TLR7 or TLR8 independently confer responsiveness to the antiviral compound R-848. Nat. Immunol. 3:499. 10.1038/ni0602-499 12032557

[35] Forsbach, A., J.-G. Nemorin, C. Montino, C. Müller, U. Samulowitz, A.P. Vicari, M. Jurk, G.K. Mutwiri, A.M. Krieg, G.B. Lipford, and J. Vollmer. 2008. Identification of RNA sequence motifs stimulating sequence-specific TLR8-dependent immune responses. J. Immunol. 180:3729–3738. 10.4049/jimmunol.180.6.3729 18322178

[36] Liu, J., C. Xu, L.-C. Hsu, Y. Luo, R. Xiang, and T.-H. Chuang. 2010. A five-amino-acid motif in the undefined region of the TLR8 ectodomain is required for species-specific ligand recognition. Mol. Immunol. 47:1083–1090. 10.1016/j.molimm.2009.11.003 PMC2815190 20004021

[37] Cervantes, J.L., B. Weinerman, C. Basole, and J.C. Salazar. 2012. TLR8: The forgotten relative revindicated. Cell. Mol. Immunol. 9:434–438. 10.1038/cmi.2012.38 PMC3498840 23085951

[38] Li, T., X. He, H. Jia, G. Chen, S. Zeng, Y. Fang, Q. Jin, and Z. Jing. 2016. Molecular cloning and functional characterization of murine toll-like receptor 8. Mol. Med. Rep. 13:1119–1126. 10.3892/mmr.2015.4668 PMC4732850 26676274

[39] Kadowaki, N., S. Ho, S. Antonenko, R.W. Malefyt, R.A. Kastelein, F. Bazan, and Y.J. Liu. 2001. Subsets of human dendritic cell precursors express different toll-like receptors and respond to different microbial antigens. J. Exp. Med. 194:863–870. 10.1084/jem.194.6.863 PMC2195968 11561001

[40] Hornung, V., S. Rothenfusser, S. Britsch, A. Krug, B. Jahrsdörfer, T. Giese, S. Endres, and G. Hartmann. 2002. Quantitative expression of toll-like receptor 1-10 mRNA in cellular subsets of human peripheral blood mononuclear cells and sensitivity to CpG oligodeoxynucleotides. J. Immunol. 168:4531–4537. 10.4049/jimmunol.168.9.4531 11970999

[41] Peng, G., Z. Guo, Y. Kiniwa, K.S. Voo, W. Peng, T. Fu, D.Y. Wang, Y. Li, H.Y. Wang, and R.-F. Wang. 2005. Toll-like receptor 8-mediated reversal of CD4+ regulatory T cell function. Science. 309:1380–1384. 10.1126/science.1113401 16123302

[42] Itoh, H., M. Tatematsu, A. Watanabe, K. Iwano, K. Funami, T. Seya, and M. Matsumoto. 2011. UNC93B1 physically associates with human TLR8 and regulates TLR8-mediated signaling. PLoS One. 6:e28500. 10.1371/journal.pone.0028500 PMC3229609 22164301

[43] Sun, H., Y. Li, P. Zhang, H. Xing, S. Zhao, Y. Song, D. Wan, and J. Yu. 2022. Targeting toll-like receptor 7/8 for immunotherapy: Recent advances and prospectives. Biomark Res. 10:89. 10.1186/s40364-022-00436-7 PMC9727882 36476317

[44] Martínez-Espinoza, I., and A. Guerrero-Plata. 2022. The relevance of TLR8 in viral infections. Pathogens. 11:134. 10.3390/pathogens11020134 PMC8877508 35215078

[45] Guiducci, C., M. Gong, A.-M. Cepika, Z. Xu, C. Tripodo, L. Bennett, C. Crain, P. Quartier, J.J. Cush, V. Pascual, . 2013. RNA recognition by human TLR8 can lead to autoimmune inflammation. J. Exp. Med. 210:2903–2919. 10.1084/jem.20131044 PMC3865472 24277153

[46] Tanji, H., U. Ohto, T. Shibata, K. Miyake, and T. Shimizu. 2013. Structural reorganization of the Toll-like receptor 8 dimer induced by agonistic ligands. Science. 339:1426–1429. 10.1126/science.1229159 23520111

[47] Tanji, H., U. Ohto, Y. Motoi, T. Shibata, K. Miyake, and T. Shimizu. 2016. Autoinhibition and relief mechanism by the proteolytic processing of Toll-like receptor 8. Proc. Natl. Acad. Sci. USA. 113:3012–3017. 10.1073/pnas.1516000113 PMC4801236 26929371

[48] Jumper, J., R. Evans, A. Pritzel, T. Green, M. Figurnov, O. Ronneberger, K. Tunyasuvunakool, R. Bates, A. Žídek, A. Potapenko, . 2021. Highly accurate protein structure prediction with AlphaFold. Nature. 596:583–589. 10.1038/s41586-021-03819-2 PMC8371605 34265844

[49] Tanji, H., U. Ohto, T. Shibata, M. Taoka, Y. Yamauchi, T. Isobe, K. Miyake, and T. Shimizu. 2015. Toll-like receptor 8 senses degradation products of single-stranded RNA. Nat. Struct. Mol. Biol. 22:109–115. 10.1038/nsmb.2943 25599397

[50] Zhang, Z., U. Ohto, T. Shibata, E. Krayukhina, M. Taoka, Y. Yamauchi, H. Tanji, T. Isobe, S. Uchiyama, K. Miyake, and T. Shimizu. 2016. Structural analysis reveals that toll-like receptor 7 is a dual receptor for guanosine and single-stranded RNA. Immunity. 45:737–748. 10.1016/j.immuni.2016.09.011 27742543

[51] Butchi, N.B., S. Pourciau, M. Du, T.W. Morgan, and K.E. Peterson. 2008. Analysis of the neuroinflammatory response to TLR7 stimulation in the brain: Comparison of multiple TLR7 and/or TLR8 agonists. J. Immunol. 180:7604–7612. 10.4049/jimmunol.180.11.7604 18490763

[52] Zheng, H., P. Wu, and P.-A. Bonnet. 2023. Recent advances on small-molecule antagonists targeting TLR7. Molecules. 28:634. 10.3390/molecules28020634 PMC9865772 36677692

[53] Petes, C., N. Odoardi, and K. Gee. 2017. The toll for trafficking: Toll-like receptor 7 delivery to the endosome. Front. Immunol. 8:1075. 10.3389/fimmu.2017.01075 PMC5591332 28928743

[54] Souyris, M., C. Cenac, P. Azar, D. Daviaud, A. Canivet, S. Grunenwald, C. Pienkowski, J. Chaumeil, J.E. Mejía, and J.-C. Guéry. 2018. *TLR7* escapes X chromosome inactivation in immune cells. Sci. Immunol. 3:eaap8855. 10.1126/sciimmunol.aap8855 29374079

[55] Shen, N., Q. Fu, Y. Deng, X. Qian, J. Zhao, K.M. Kaufman, Y.L. Wu, C.Y. Yu, Y. Tang, J.-Y. Chen, . 2010. Sex-specific association of X-linked Toll-like receptor 7 (TLR7) with male systemic lupus erythematosus. Proc. Natl. Acad. Sci. USA. 107:15838–15843. 10.1073/pnas.1001337107 PMC2936646 20733074

[56] Youness, A., C. Cenac, B. Faz-López, S. Grunenwald, F.J. Barrat, J. Chaumeil, J.E. Mejía, and J.-C. Guéry. 2023. TLR8 escapes X chromosome inactivation in human monocytes and CD4^+^ T cells. Biol. Sex Differ. 14:60. 10.1186/s13293-023-00544-5 PMC10506212 37723501

[57] Tojo, S., Z. Zhang, H. Matsui, M. Tahara, M. Ikeguchi, M. Kochi, M. Kamada, H. Shigematsu, A. Tsutsumi, N. Adachi, . 2020. Structural analysis reveals TLR7 dynamics underlying antagonism. Nat. Commun. 11:5204. 10.1038/s41467-020-19025-z PMC7562955 33060576

[58] Kaushik, D., A. Kaur, N. Petrovsky, and D.B. Salunke. 2021. Structural evolution of toll-like receptor 7/8 agonists from imidazoquinolines to imidazoles. RSC Med. Chem. 12:1065–1120. 10.1039/d1md00031d PMC8292987 34355178

[59] Greulich, W., M. Wagner, M.M. Gaidt, C. Stafford, Y. Cheng, A. Linder, T. Carell, and V. Hornung. 2019. TLR8 is a sensor of RNase T2 degradation products. Cell. 179:1264–1275.e13. 10.1016/j.cell.2019.11.001 PMC7116005 31778653

[60] Hu, T., S.R. Suter, M.M. Mumbleau, and P.A. Beal. 2018. TLR8 activation and inhibition by guanosine analogs in RNA: Importance of functional groups and chain length. Bioorg. Med. Chem. 26:77–83. 10.1016/j.bmc.2017.11.020 PMC5733708 29174509

[61] Alharbi, A.S., S. Sapkota, Z. Zhang, R. Jin, E. Rupasinghe, W.S.N. Jayasekara, D. Yu, M. Speir, L. Wilkinson-White, L. Cubeddu, . 2026. 2′-O-Methyl-guanosine RNA fragments antagonize TLR7 and TLR8 to limit autoimmunity. Nat. Immunol. 27:1–14. 10.1038/s41590-026-02429-2 PMC13043311 41667621

[62] Krogh, N., M.D. Jansson, S.J. Häfner, D. Tehler, U. Birkedal, M. Christensen-Dalsgaard, A.H. Lund, and H. Nielsen. 2016. Profiling of 2’-O-Me in human rRNA reveals a subset of fractionally modified positions and provides evidence for ribosome heterogeneity. Nucleic Acids Res. 44:7884–7895. 10.1093/nar/gkw482 PMC5027482 27257078

[63] Krüger, A., M. Oldenburg, C. Chebrolu, D. Beisser, J. Kolter, A.M. Sigmund, J. Steinmann, S. Schäfer, H. Hochrein, S. Rahmann, . 2015. Human TLR8 senses UR/URR motifs in bacterial and mitochondrial RNA. EMBO Rep. 16:1656–1663. 10.15252/embr.201540861 PMC4687425 26545385

[64] Sakaniwa, K., and T. Shimizu. 2020. Targeting the innate immune receptor TLR8 using small-molecule agents. Acta Crystallogr. D Struct. Biol. 76:621–629. 10.1107/S2059798320006518 PMC7336380 32627735

[65] Desnues, B., A.B. Macedo, A. Roussel-Queval, J. Bonnardel, S. Henri, O. Demaria, and L. Alexopoulou. 2014. TLR8 on dendritic cells and TLR9 on B cells restrain TLR7-mediated spontaneous autoimmunity in C57BL/6 mice. Proc. Natl. Acad. Sci. USA. 111:1497–1502. 10.1073/pnas.1314121111 PMC3910605 24474776

[66] Crow, Y.J., and D.B. Stetson. 2022. The type I interferonopathies: 10 years on. Nat. Rev. Immunol. 22:471–483. 10.1038/s41577-021-00633-9 PMC8527296 34671122

[67] Hatakeyama, C., C.L. Anderson, C.L. Beever, M.S. Peñaherrera, C.J. Brown, and W.P. Robinson. 2004. The dynamics of X-inactivation skewing as women age. Clin. Genet. 66:327–332. 10.1111/j.1399-0004.2004.00310.x 15355435

[68] Zito, A., M.N. Davies, P.-C. Tsai, S. Roberts, R. Andres-Ejarque, S. Nardone, J.T. Bell, C.C.Y. Wong, and K.S. Small. 2019. Heritability of skewed X-inactivation in female twins is tissue-specific and associated with age. Nat. Commun. 10:5339. 10.1038/s41467-019-13340-w PMC6877649 31767861

[69] Schmitz, E.G., A.J. Paul, R. Ghosh, N. Saucier, A. Kolicheski, S.I. Risma, K.P. McDaniels, M. Liu, K.L. Lewis, A.A. de Jesus, . 2026. Targeted deep sequencing identifies mosaicism in patients with immune dysregulation. J. Allergy Clin. Immunol. 157:1179–1194. 10.1016/j.jaci.2026.01.023 PMC12916128 41654260

[70] Li, L., X. Liu, K.L. Sanders, J.L. Edwards, J. Ye, F. Si, A. Gao, L. Huang, E.C. Hsueh, D.A. Ford, . 2019. TLR8-Mediated metabolic control of human Treg function: A mechanistic target for cancer immunotherapy. Cell Metab. 29:103–123.e5. 10.1016/j.cmet.2018.09.020 PMC7050437 30344014

[71] Geginat, J., A. Lanzavecchia, and F. Sallusto. 2003. Proliferation and differentiation potential of human CD8+ memory T-cell subsets in response to antigen or homeostatic cytokines. Blood. 101:4260–4266. 10.1182/blood-2002-11-3577 12576317

[72] D’Asaro, M., F. Dieli, N. Caccamo, M. Musso, F. Porretto, and A. Salerno. 2006. Increase of CCR7− CD45RA+ CD8 T cells (T(EMRA)) in chronic graft-versus-host disease. Leukemia. 20:545–547. 10.1038/sj.leu.2404079 16408100

[73] Neirinck, J., M. Buysse, C. De Vriendt, M. Hofmans, and C. Bonroy. 2025. The role of immunophenotyping in common variable immunodeficiency: A narrative review. Crit. Rev. Clin. Lab. Sci. 62:65–84. 10.1080/10408363.2024.2404842 39364936

[74] Brouet, J.-C., A. Chedeville, J.P. Fermand, and B. Royer. 2000. Study of the B cell memory compartment in common variable immunodeficiency. Eur. J. Immunol. 30:2516–2520. 10.1002/1521-4141(200009)30:9<2516::AID-IMMU2516>3.0.CO;2-Z 11009084

[75] Warnatz, K., C. Wehr, R. Dräger, S. Schmidt, H. Eibel, M. Schlesier, and H.-H. Peter. 2002. Expansion of CD19(hi)CD21(lo/neg) B cells in common variable immunodeficiency (CVID) patients with autoimmune cytopenia. Immunobiology. 206:502–513. 10.1078/0171-2985-00198 12607725

[76] Kawai, T., and S. Akira. 2006. TLR signaling. Cell Death Differ. 13:816–825. 10.1038/sj.cdd.4401850 16410796

[77] Heinz, L.X., J. Lee, U. Kapoor, F. Kartnig, V. Sedlyarov, K. Papakostas, A. César-Razquin, P. Essletzbichler, U. Goldmann, A. Stefanovic, . 2020. TASL is the SLC15A4-associated adaptor for IRF5 activation by TLR7-9. Nature. 581:316–322. 10.1038/s41586-020-2282-0 PMC7610944 32433612

[78] Lainšček, D., S. Horvat, K. Dolinar, F. Ivanovski, R. Romih, S. Pirkmajer, R. Jerala, and M. Manček-Keber. 2024. MyD88 protein destabilization mitigates NF-κB-dependent protection against macrophage apoptosis. Cell Commun. Signal. 22:549. 10.1186/s12964-024-01930-1 PMC11568545 39550582

[79] Robbins, S.H., T. Walzer, D. Dembélé, C. Thibault, A. Defays, G. Bessou, H. Xu, E. Vivier, M. Sellars, P. Pierre, . 2008. Novel insights into the relationships between dendritic cell subsets in human and mouse revealed by genome-wide expression profiling. Genome Biol. 9:R17. 10.1186/gb-2008-9-1-r17 PMC2395256 18218067

[80] Assil, S., S. Coléon, C. Dong, E. Décembre, L. Sherry, O. Allatif, B. Webster, and M. Dreux. 2019. Plasmacytoid dendritic cells and infected cells form an interferogenic synapse required for antiviral responses. Cell Host Microbe. 25:730–745.e6. 10.1016/j.chom.2019.03.005 31003939

[81] Ehlers, C., T. Thiele, H. Biermann, S. Traidl, L. Bruns, A. Ziegler, M. Schefzyk, L.M. Bartsch, U. Kalinke, T. Witte, and T. Graalmann. 2025. Toll-like receptor 8 is expressed in monocytes in contrast to plasmacytoid dendritic cells and mediates aberrant interleukin-10 responses in patients with systemic sclerosis. Arthritis Rheumatol. 77:59–66. 10.1002/art.42964 PMC11685002 39112920

[82] Berg, R.E., E. Crossley, S. Murray, and J. Forman. 2003. Memory CD8+ T cells provide innate immune protection against *Listeria monocytogenes* in the absence of cognate antigen. J. Exp. Med. 198:1583–1593. 10.1084/jem.20031051 PMC1592647 14623912

[83] Chowdhury, F.Z., H.J. Ramos, L.S. Davis, J. Forman, and J.D. Farrar. 2011. IL-12 selectively programs effector pathways that are stably expressed in human CD8+ effector memory T cells in vivo. Blood. 118:3890–3900. 10.1182/blood-2011-05-357111 PMC3193266 21832277

[84] Freeman, B.E., E. Hammarlund, H.-P. Raué, and M.K. Slifka. 2012. Regulation of innate CD8+ T-cell activation mediated by cytokines. Proc. Natl. Acad. Sci. USA. 109:9971–9976. 10.1073/pnas.1203543109 PMC3382521 22665806

[85] Goplen, N.P., V. Saxena, K.M. Knudson, A.G. Schrum, D. Gil, M.A. Daniels, R. Zamoyska, and E. Teixeiro. 2016. IL-12 signals through the TCR to support CD8 innate immune responses. J. Immunol. 197:2434–2443. 10.4049/jimmunol.1600037 PMC5010952 27521342

[86] Lutz, V., V.M. Hellmund, F.S.R. Picard, H. Raifer, T. Ruckenbrod, M. Klein, T. Bopp, R. Savai, P. Duewell, C.U. Keber, . 2023. IL18 receptor signaling regulates tumor-reactive CD8+ T-cell exhaustion via activation of the IL2/STAT5/mTOR pathway in a pancreatic cancer model. Cancer Immunol. Res. 11:421–434. 10.1158/2326-6066.CIR-22-0398 36758176

[87] Antonelli, A., S.M. Ferrari, P. Fallahi, S. Frascerra, E. Santini, S.S. Franceschini, and E. Ferrannini. 2009. Monokine induced by interferon gamma (IFNgamma) (CXCL9) and IFNgamma inducible T-cell alpha-chemoattractant (CXCL11) involvement in Graves’ disease and ophthalmopathy: Modulation by peroxisome proliferator-activated receptor-gamma agonists. J. Clin. Endocrinol. Metab. 94:1803–1809. 10.1210/jc.2008-2450 19276231

[88] Van Raemdonck, K., P.E. Van den Steen, S. Liekens, J. Van Damme, and S. Struyf. 2015. CXCR3 ligands in disease and therapy. Cytokine Growth Factor. Rev. 26:311–327. 10.1016/j.cytogfr.2014.11.009 25498524

[89] Bhat, P., G. Leggatt, N. Waterhouse, and I.H. Frazer. 2017. Interferon-γ derived from cytotoxic lymphocytes directly enhances their motility and cytotoxicity. Cell Death Dis. 8:e2836. 10.1038/cddis.2017.67e2836-e2836 PMC5520949 28569770

[90] Mackay, F., and C.R. Mackay. 2002. The role of BAFF in B-cell maturation, T-cell activation and autoimmunity. Trends Immunol. 23:113–115. 10.1016/s1471-4906(01)02159-7 11864827

[91] Marchand, T., C. Pastoret, A. Moignet, M. Roussel, and T. Lamy. 2024. Large granular lymphocyte leukemia: A clonal disorder with autoimmune manifestations. Hematol. Am. Soc. Hematol. Educ. Program. 2024:143–149. 10.1182/hematology.2024000539 PMC11665628 39644019

[92] Lin, F.-c., M. Karwan, B. Saleh, D.L. Hodge, T. Chan, K.C. Boelte, J.R. Keller, and H.A. Young. 2014. IFN-γ causes aplastic anemia by altering hematopoietic stem/progenitor cell composition and disrupting lineage differentiation. Blood. 124:3699–3708. 10.1182/blood-2014-01-549527 PMC4263980 25342713

[93] Selleri, C., J.P. Maciejewski, T. Sato, and N.S. Young. 1996. Interferon-gamma constitutively expressed in the stromal microenvironment of human marrow cultures mediates potent hematopoietic inhibition. Blood. 87:4149–4157. 8639773

[94] Lamy, T., A. Moignet, and T.P. Loughran Jr. 2017. LGL leukemia: From pathogenesis to treatment. Blood. 129:1082–1094. 10.1182/blood-2016-08-692590 28115367

[95] Marchand, T., T. Lamy, and T.P. Loughran Jr. 2024. A modern view of LGL leukemia. Blood. 144:1910–1923. 10.1182/blood.2023021790 38848524

[96] Liu, J.H., S. Wei, T. Lamy, P.K. Epling-Burnette, G. Starkebaum, J.Y. Djeu, and T.P. Loughran. 2000. Chronic neutropenia mediated by Fas ligand. Blood. 95:3219–3222. 10807792

[97] Schmitz, E.G., M. Griffith, O.L. Griffith, and M.A. Cooper. 2025. Identifying genetic errors of immunity due to mosaicism. J. Exp. Med. 222:e20241045. 10.1084/jem.20241045 PMC11998702 40232243

[98] Zhang, S., Z. Hu, H. Tanji, S. Jiang, N. Das, J. Li, K. Sakaniwa, J. Jin, Y. Bian, U. Ohto, . 2018. Small-molecule inhibition of TLR8 through stabilization of its resting state. Nat. Chem. Biol. 14:58–64. 10.1038/nchembio.2518 PMC5726935 29155428

[99] Tanaka, Y., A. Kumanogoh, T. Atsumi, T. Ishii, F. Tago, M. Aoki, S. Yamamuro, and S. Akira. 2024. Safety, pharmacokinetics, biomarker response and efficacy of E6742: A dual antagonist of toll-like receptors 7 and 8, in a first in patient, randomised, double-blind, phase I/II study in systemic lupus erythematosus. RMD Open. 10:e004701. 10.1136/rmdopen-2024-004701 PMC11409405 39289029

[100] Morand, E.F., V.P. Werth, J. Wenzel, R. Furie, M. Dall’Era, J. Sanchez-Guerrero, S. Roy, S.G. Goodson, F. Moreau, H. Gühring, . 2026. Efficacy and safety of enpatoran, a toll-like receptor 7/8 inhibitor, in patients with skin manifestations of cutaneous lupus erythematosus or systemic lupus erythematosus: Findings from cohort A of a multicentre, international, double-blind, placebo-controlled, dose-finding phase 2 trial. Lancet Rheumatol. 8:e513–e527. 10.1016/S2665-9913(25)00337-6 42107375

[101] Beeline Medicines Corporation . 2026. A Phase 2, Multicenter, Randomized, Double-blind, Placebo-Controlled, Study to Evaluate the Efficacy and Safety of Afimetoran in Participants With Active Systemic Lupus Erythematosus. NCT04895696.

[102] Kawai, T., S. Sato, K.J. Ishii, C. Coban, H. Hemmi, M. Yamamoto, K. Terai, M. Matsuda, J.-i Inoue, S. Uematsu, . 2004. Interferon-alpha induction through Toll-like receptors involves a direct interaction of IRF7 with MyD88 and TRAF6. Nat. Immunol. 5:1061–1068. 10.1038/ni1118 15361868

[103] Feng, Y., C. Chen, A. Shao, L. Wu, H. Hu, and T. Zhang. 2024. Emerging interleukin-1 receptor-associated kinase 4 (IRAK4) inhibitors or degraders as therapeutic agents for autoimmune diseases and cancer. Acta Pharm. Sin B. 14:5091–5105. 10.1016/j.apsb.2024.09.008 PMC11725142 39807338

[104] Parrondo, R.D., M. Iqbal, R. Von Roemeling, C. Von Roemeling, and H.W. Tun. 2023. IRAK-4 inhibition: Emavusertib for the treatment of lymphoid and myeloid malignancies. Front. Immunol. 14:1239082. 10.3389/fimmu.2023.1239082 PMC10637517 37954584

[105] Jodl, S.J., W. Ten Voorde, S. Klein, A. Wagenfeld, F.S. Zollmann, M. Feldmüller, N.B. Klarenbeek, D.T. de Bruin, M.A.A. Jansen, R. Rissmann, . 2024. The oral IRAK4 inhibitors zabedosertib and BAY1830839 suppress local and systemic immune responses in a randomized trial in healthy male volunteers. Clin. Transl Sci. 17:e13771. 10.1111/cts.13771 PMC10955609 38511583

[106] Al-Azab, M., E. Idiiatullina, Z. Liu, M. Lin, K. Hrovat-Schaale, H. Xian, J. Zhu, M. Yang, B. Lu, Z. Zhao, . 2024. Genetic variants in UNC93B1 predispose to childhood-onset systemic lupus erythematosus. Nat. Immunol. 25:969–980. 10.1038/s41590-024-01846-5 PMC11147776 38831104

[107] Gantier, M.P., A.T. Irving, M. Kaparakis-Liaskos, D. Xu, V.A. Evans, P.U. Cameron, J.A. Bourne, R.L. Ferrero, M. John, M.A. Behlke, and B.R.G. Williams. 2010. Genetic modulation of TLR8 response following bacterial phagocytosis. Hum. Mutat. 31:1069–1079. 10.1002/humu.21321 20652908

[108] Oh, D.-Y., S. Taube, O. Hamouda, C. Kücherer, G. Poggensee, H. Jessen, J.K. Eckert, K. Neumann, A. Storek, M. Pouliot, . 2008. A functional toll-like receptor 8 variant is associated with HIV disease restriction. J. Infect. Dis. 198:701–709. 10.1086/590431 18605904

[109] Chen, S., L.C. Francioli, J.K. Goodrich, R.L. Collins, M. Kanai, Q. Wang, J. Alföldi, N.A. Watts, C. Vittal, L.D. Gauthier, . 2024. A genomic mutational constraint map using variation in 76,156 human genomes. Nature. 625:92–100. 10.1038/s41586-023-06045-0 PMC11629659 38057664

[110] The 1000 Genomes Project Consortium . 2015. A global reference for human genetic variation. Nature. 526:68–74. 10.1038/nature15393 PMC4750478 26432245

